# Using Hybrid MnO_2_-Au Nanoflowers to Accelerate ROS Scavenging and Wound Healing in Diabetes

**DOI:** 10.3390/pharmaceutics16101244

**Published:** 2024-09-25

**Authors:** Ning Jiang, Xinwei Liu, Baiyan Sui, Jiale Wang, Xin Liu, Zun Zhang

**Affiliations:** 1 Department of Oral and Craniomaxillofacial Science, Shanghai Key Laboratory of Stomatology, Shanghai Ninth People’s Hospital, School of Medicine, College of Stomatology, Shanghai Jiao Tong University, Shanghai 200011, China; jiangning@angelalign.com; 2 Department of Dental Materials, Shanghai Biomaterials Research & Testing Center, Shanghai Ninth People’s Hospital, Shanghai Jiao Tong University School of Medicine, College of Stomatology, Shanghai Jiao Tong University, Shanghai 200011, China; liuxinweiiii@sjtu.edu.cn (X.L.); baiyan.sui@shsmu.edu.cn (B.S.); 3 National Center for Stomatology, National Clinical Research Center for Oral Diseases, Shanghai Key Laboratory of Stomatology, Shanghai Jiao Tong University, Shanghai 200011, China; 4 College of Science, Donghua University, Shanghai 201620, China; jiale.wang@dhu.edu.cn; 5 Shanghai Institute of Intelligent Electronics and Systems, Donghua University, Shanghai 201620, China; 6 Department of Stomatology, Shanghai East Hospital, Tongji University, Shanghai 200120, China

**Keywords:** MnO_2_-Au nanoflowers, nanoenzyme catalysis, ROS elimination, wound regeneration, diabetes

## Abstract

**Objectives:** Excessive reactive oxygen species (ROS) in diabetic wounds are major contributors to chronic wounds and impaired healing, posing significant challenges in regenerative medicine. Developing innovative drug delivery systems is crucial to address these issues by modifying the adverse microenvironment and promoting effective wound healing. **Methods:** Herein, we designed a novel drug delivery platform using manganese dioxide nanoflower hybridized gold nanoparticle composites (MnO_2_-Au) synthesized via a hydrothermal reaction, and investigated the potential of MnO_2_-Au nanoflowers to relieve the high oxidative stress microenvironment and regulate diabetic wound tissue healing. **Results:** This hybrid material demonstrated superior catalytic activity compared to MnO_2_ alone, enabling the rapid decomposition of hydrogen peroxide and a substantial reduction in ROS levels within dermal fibroblasts. The MnO_2_-Au nanoflowers also facilitated enhanced dermal fibroblast migration and *Col-I* expression, which are critical for tissue regeneration. Additionally, a hydrogel-based wound dressing incorporating MnO_2_-Au nanoflowers was developed, showing its potential as an intelligent drug delivery system. This dressing significantly reduced oxidative stress, accelerated wound closure, and improved the quality of neonatal epithelial tissue regeneration in a diabetic rat skin defect model. **Conclusions:** Our findings underscore the potential of MnO_2_-Au nanoflower-based drug delivery systems as a promising therapeutic approach for chronic wound healing, particularly in regenerative medicine.

## 1. Introduction

Globally, the prevalence of diabetes mellitus exceeds half a billion people, representing a severe chronic health condition [1]. Diabetic ulcers are typical wounds in diabetic patients that significantly affect their quality of life, and are characterized by their prolonged inability to heal and a significant cause of nontraumatic amputations in diabetes patients [2,3]. Among the multilevel mechanisms of difficult wound healing in diabetes patients, reactive oxygen species (ROS) overload plays a critical role [4]. Hyperglycemia exacerbates the inflammatory response by generating large amounts of ROS during metabolism. Afterward, the inflammatory process triggers the synthesis of various pro-inflammatory cytokines that increase the generation of ROS [5,6]. The impaired epithelial function and depressed cell activity from the oxidative stress microenvironment substantially impede wound closure and skin regeneration [4]. Consequently, the construction of wound repair materials to scavenge excessive ROS is a promising solution to address the difficult clinical problem of diabetic ulcer recuperation.

During the early stages of diabetic wounds, ROS levels, including hydrogen peroxide (H_2_O_2_) and superoxide anion (O^−2^), increase without an associated surge in the activity of the corresponding antioxidant enzymes [7]. Excess ROS disrupts protein molecules, nucleic acids, lipids, and cellular components, negatively affecting angiogenesis and fibroblast migration [8,9]. The high ROS concentration can also trigger inflammatory cell migration into the wound and destroy the extracellular matrix (ECM) components. An impaired ECM results in prolonged healing and augmented susceptibility to bacterial invasion [10]. Extensive attention and research have been focused on the development and construction of nanoenzyme wound repair dressings, which can reduce the surge in ROS from localized diabetic wounds to promote wound regeneration [11,12,13]. Nanoenzymatic materials with high catalase, superoxide dismutase, and glutathione peroxidase have the advantages of eliminating ROS, reducing the inflammatory response of wounds, promoting wound angiogenesis, and accelerating tissue repair and regeneration [14,15,16,17]. Meanwhile, in contrast to the low stability and complex storage of natural enzymes, nanoenzymes exhibit stable catalytic properties at low doses, which considerably responds to the requirement for chronic wound healing.

Manganese dioxide (MnO_2_) nanomaterials are one of the most effective nanoenzymes due to the abundance of oxygen vacancies that can efficiently bind to free radicals, and MnO_2_ nanomaterials can catalyze the decomposition of the most abundant endogenous H_2_O_2_ into O_2_, leading to the alleviation of oxidative stress [18,19]. The study by Cao et al. showed that the MnO_2_ nanoenzyme-based complex methacrylate anhydride gelatin (GelMA) could eliminate the intracellular overexpression of ROS and reverse the inflammatory microenvironment to promote diabetic wound healing [20]. In addition, MnO_2_ nanomaterials have excellent controlled biodegradation properties and biosafety compared with other nanoenzymes such as cerium oxide (CeO_2_) nanoclusters, gold nanoclusters, and C3N4 nanofusiform [21,22]. However, nanoenzymes derived from catalytic materials based on metal oxides are limited primarily by the high valence of the metal center, which is attributed to the coordinated electronegative atoms. Owing to the imbalance in valence phases, there are still significant challenges in developing long-term catalytic metal oxide nanoenzymes with ROS scavenging properties and reversible reductive cycling [23]. Gold nanoparticles (Au NPs) possess a favorable surface plasmonic resonance (SPR) effect. It has been recently reported that SPR excitation in plasmonic materials can strengthen catalytic processes [24,25]. Our previous study also confirmed that the electrons generated in a hybrid nanomaterial composed of MnO_2_ nanoflowers decorated with Au NPs (MnO_2_-Au) can be delivered to the Au NPs, increasing the electron density in Au and promoting SPR-mediated activities [26]. These findings show that the electron-regulated strategy enhances the ROS cleavage ability and that nanoenzymatic systems constructed with Au NPs decorated with MnO_2_ with SPR effects can fulfill this purpose.

In this study, we investigated the potential of MnO_2_-Au nanoflowers as nanoenzymes to relieve the high oxidative stress microenvironment of local wounds, and we studied their performance in regulating diabetic wound tissue healing. Hydrogen peroxide could be rapidly decomposed by MnO_2_-Au nanoflowers within 2 h, effectively reducing ROS levels in dermal fibroblasts, and thereby promoting the migration and *Col-I* expression of the cells. Furthermore, the MnO_2_-Au nanoflower composite hydrogel enhanced the percentage of wound closure and the healing of neuroepithelial tissue with significant collagen deposition. These findings highlight that MnO_2_-Au nanoflowers could be potentially applied as a nanoenzyme-based drug delivery system for promoting diabetic wound healing.

## 2. Materials and Methods

### 2.1. Synthesis of MnO_2_ Nanoflowers

According to a previous study, MnO_2_ nanoflowers were synthesized using a hydrothermal reaction [26]. Briefly, 1.0 g of KMnO_4_ (Sinopharm, Shanghai, China) and 0.4 g of MnSO_4_ (Sinopharm, Shanghai, China) powder were mixed in 30 mL of deionized water under continuous stirring and transferred to a Teflon-lined stainless-steel autoclave (Yikai Instrument Equipment Co., Ltd, Shanghai, China). The autoclave was then heated and stirred at 140 °C for 1 h. Then, it was allowed to cool to room temperature. Then, the obtained MnO_2_ nanoflowers were washed and dried for the following experiments.

### 2.2. Synthesis of MnO_2_ Nanoflowers Decorated with Au NPs (MnO_2_-Au)

First, 100 mg of MnO_2_ nanoflowers was added to 100 mL of deionized water and dissolved by ultrasonication to obtain a homogeneous solution. Then, 140 mg of PVP powder (Merck, Darmstadt, Germany) and 20 mg of sodium citrate (Sinopharm, Shanghai, China) were added, followed by heating to boiling under continuous stirring. Afterward, 200 mM/L of HAuCl_4_ (aq) (Merck, Darmstadt, Germany) was added to the reaction system and boiled for 1 h. Finally, the obtained MnO_2_ nanoflowers were washed and dried for the following experiments.

### 2.3. Characterization of MnO_2_ and MnO_2_-Au Nanoflowers

The morphology and elemental composition of MnO_2_ and MnO_2_-Au nanoflowers were observed and analyzed using a field emission scanning electron microscope (FE-SEM, Hitachi, Tokyo, Japan) equipped with energy dispersive X-ray spectrometry (EDS). The microstructures of MnO_2_ and MnO_2_-Au nanoflowers were recorded using a double-corrected microscope JEM-ARM300F (GrandARM, JEOL, Tokyo, Japan). The zeta potential of two MnO_2_ nanoflowers in PBS was measured by a Zetasizer Nano ZS 90 (Malvern Instruments Ltd., Worcestershire, UK).

### 2.4. Catalase-Mimicking Activity

We examined the hydrogen peroxide scavenging efficiency using a hydrogen peroxide assay kit (Beyotime, Shanghai, China) to assess the catalase-mimicking activity of the two nanoflowers. The MnO_2_ and MnO_2_-Au nanoflowers (1 mg/mL) were mixed with phosphate buffer (25 mM, pH 7.4) containing 1 of mM H_2_O_2_ (Sinopharm, Shanghai, China). The supernatant was collected at different time points (1, 2, 24, and 48 h), and 100 μL of hydrogen peroxide detection reagent was mixed with 50 μL of supernatant. After 30 min of incubation at room temperature, the results were recorded under the absorbance at 560 nm via a Multiskan FC microplate photometer (Thermo, MA, USA), and the concentration of hydrogen peroxide was calculated to evaluate its scavenging and the catalase-mimicking activity of MnO_2_ and MnO_2_-Au nanoflowers.

### 2.5. In Vitro Cell Viability Assay

Human dermal fibroblasts (HDFs, Cat. No. 2320) were obtained from ScienCell (CA, USA), and the cells were cultured in fibroblast medium (Cat. No. 2301, ScienCell, CA, USA) containing 2% fetal bovine serum (FBS), 1% fibroblast growth supplement (FGS), streptomycin (100 g/L), and penicillin (100 U/mL) at 37 °C in a humidified incubator equilibrated with 5% CO_2_.

An in vitro simulated medium (SM) was designed to mimic the oxidative stress microenvironment and the corresponding protective responses of the nanoflowers. As an active type of ROS, H_2_O_2_-treated culture media were selected to simulate the diabetic wound microenvironment, which involves excessive oxidative stress in the ECM. MnO_2_ or MnO_2_-Au nanoflowers (1 mg/mL) were added to media containing 500 μmol/L H_2_O_2_, and the nanoflowers were removed after incubation for 1 h at 37 °C. The remaining cell culture medium is referred to as the SM of the corresponding nanoflowers, the H_2_O_2_-containing medium without material treatment was set as a positive control (PC), and the cell medium was set as a negative control (NC). The HDF were seeded in a 96-well plate at a density of 5 × 10^3^ cells per well and incubated for 24 h. Then, the NC group, PC group, MnO_2_ group, and MnO_2_-Au group were added to the 96-well plate (100 μL per well) and incubated for 24 h, respectively. The cell viability of the oxidized HDFs was correlated with the staining result using a Cell Counting Kit-8 assay (CCK-8, Beyotime, Shanghai, China).

### 2.6. Intracellular ROS Scavenging Assay

The intracellular ROS scavenging properties of MnO_2_ and MnO_2_-Au nanoflowers were examined using a ROS-sensitive probe (Beyotime, Shanghai, China). HDFs were seeded in 24-well plates at a density of 8 × 10^4^ cells per well. After 24 h of incubation, the HDFs were loaded with a 2′,7′-Dichlorodihydrofluorescein diacetate (DCFH–DA) working solution, incubated at 37 °C in the dark for 30 min, and washed with phosphate buffer (25 mM, pH 7.4) in triplicate. Then, the NC group, PC group, MnO_2_ group, and MnO_2_-Au group were added to the 24-well plate (500 μL per well) and incubated. The level of oxidative stress in the HDF cells was determined via flow cytometry (FACSCelesta, Franklin Lakes, BD, NJ, USA), and the fluorescence images were captured after 30 min of co-culture using Agilent BioTek Cytation 3 (Biotek, CA, USA).

### 2.7. Cell Migration and Collagen I Expression

Transwell chambers (Corning, MA, USA) with 8.0 µm-pore polycarbonate filters were used in the migration assay. HDF cells were seeded in the upper chamber with 200 μL of serum-free medium at a density of 1 × 10^4^ cells, and the lower chamber was filled with 600 μL of SM derived from the different groups pre-treated for 1 h at 37 °C, including MnO_2_ or MnO_2_-Au nanoflowers (1 mg/mL) co-incubated with 500 μmol/L of H_2_O_2_, and 500 μmol/L of H_2_O_2_ alone (set as control). After they were stimulated with the treated media for 6 and 24 h, the migration result of the HDF cells was recorded from the microscope.

Real-time quantitative reverse transcription polymerase chain reaction (RT-qPCR) was performed to evaluate the collagen I expression. HDF cells were seeded in a 24-well plate at a density of 7 × 10^4^ cells/mL and then incubated with 1 mL of SM derived from the different groups for 24 h, including MnO_2_ or MnO_2_-Au nanoflowers (1 mg/mL) co-incubated with 500 μmol/L of H_2_O_2_ pre-treated for 1 h at 37 °C, and 500 μmol/L of H_2_O_2_ alone pre-treated for 1 h at 37 °C (set as control). The total RNA of the HDF cells was extracted using a RNeasy Mini Kit (Qiagen, Hilden, Germany). The gene expression of collagen type I alpha 1 chain (*COL1A1*) was detected, and *GAPDH* was set as a housekeeping gene. Table 1 shows the primer sequences of HDFs used in the gene expression experiment.

### 2.8. Preparation of MnO_2_ and MnO_2_-Au Nanoflower Composite Hydrogels

To further validate the feasibility of MnO_2_ and MnO_2_-Au nanoflowers in targeting diabetic skin damage, we prepared a composite hydrogel loaded with nanoflowers. Briefly, 4-arm-poly (ethylene glycol) NHS (4-arm-PEG-NHS) and 4-arm-poly (ethylene glycol) amines (4-arm-PEG-NH_2_) were obtained from EngineeringforLife Group (Soochow, China). MnO_2_ and MnO_2_-Au nanoflowers were dispersed in PBS at 0.5 mg/mL. Then, 4-arm-PEG NHS and NH_2_ powders were dissolved in PBS containing nanoflowers at 4% (*w*/*v*). The 4-arm-PEG-NHS and 4-arm-PEG-NH_2_ solutions containing nanoflowers were co-mixed using a duplex injector, and the nanoflower composite hydrogels (MnO_2_ hydrogel and MnO_2_-Au hydrogel) were obtained by curing for 120 s at room temperature and used for the following experiments.

### 2.9. Characterization and Cell Viability of MnO_2_ and MnO_2_-Au Nanoflower Composite Hydrogels

SEM-EDS mapping (Mira3, Tescan, Kohoutovice, Czech Republic) was employed to observe and analyze the morphology of the samples and the distribution of nanoflowers in the composite hydrogels. To assess the releasability of nanoflowers from the composite hydrogel, we immersed 100 μL of cured composited hydrogel in 1 mL of PBS at 37 °C for 14 days, and the released nanoflowers were measured using inductively coupled plasma mass spectrometry (ICP-MS, Agilent 7700s, Biotek, CA, USA) at 7 and 14 days. The efficiency of hydrogen peroxide scavenging was assayed to evaluate the catalytic-mimicking activity of the composite hydrogel containing the nanoflowers. Briefly, 100 μL of the composite hydrogel was mixed with phosphate buffer (25 mmol, pH 7.4) containing 1 mmol of H_2_O_2_. The supernatant was collected and measured according to the method in Section 2.4. Cell viability was determined using a CCK-8 kit (Beyotime, Shanghai, China). Briefly, mouse-derived NCTC clone 929 (L 929) cells were from Cell Bank, Chinese Academy of Sciences (Shanghai, China), and the cells were cultured in MEM medium (Cat. No. 11095080, Gibco, MD, USA) supplemented with 10% FBS (SH30070.03, Cytiva, MA, USA), 1% penicillin and streptomycin (SV30010, Cytiva, MA, USA) at 37 °C containing 5% CO_2_. L-929 cells were seeded onto the plates with or without hydrogels. After culturing for 24 h, the cell viability was determined by detecting the absorbance at 450 nm for each group.

### 2.10. Animals and Skin Defect Model

Male C57BL/6J-db/db mice (6 weeks, 36–40 g) at 8 weeks to 10 weeks of age were obtained from the Shanghai Model Organisms Center (Shanghai, China). The animal study protocol was approved by the Independent Ethics Committee of Shanghai Ninth People’s Hospital, Shanghai Jiao Tong University School of Medicine (protocol code: SH9H-2022-A417-SB). The db/db mouse represents a proven animal model of type 2 diabetes mellitus that exhibits persistent hyperinsulinemia and high plasma glucose levels. The dorsal hair of the mice was shaved, and a full-thickness skin wound with a diameter of 10 mm was made on the back of each mouse to construct a diabetic skin defect model. The animals were randomly divided into four groups (n = 4): Bank, Gel, MnO_2_ Gel, and MnO_2_-Au Gel. The wounds were observed and analyzed using ImageJ at 0, 3, 7, and 14 days.

### 2.11. Histological Observation and Evaluation of the ROS Profile

At the end of the 14-day animal experiment, wounds and adjacent normal skin were collected and fixed in 4% paraformaldehyde. The wound tissues were observed using H&E and Masson’s trichrome staining to analyze the regenerative effects induced by MnO_2_ and the MnO_2_-Au-based hydrogel. The collagen occupied area ratio of the histological section was analyzed using Image-J (version 1.50d). The skin sections were incubated in dihydroethidium (DHE) (Beyotime, Shanghai, China) and Dapi dyes (Beyotime, Shanghai, China) to observe the change in oxidative stress in the different groups.

### 2.12. Statistical Analysis

Data are presented as the mean ± standard deviation. SPSS 20.0 software was used for statistical analysis. The statistical comparisons were established using the one-way ANOVA or Student’s *t*-test. Statistically significant outcomes are indicated by a *p* value less than 0.05. The data were demonstrated with * for 0.01 < *p* < 0.05, ** for 0.001 < *p* < 0.01, and *** for *p* < 0.001. Details regarding sample sizes (n) and probability values are shown in the corresponding figure captions.

## 3. Results and Discussion

### 3.1. Synthesis and Characterization of MnO_2_ and MnO_2_-Au Nanoflowers

**Figure 1:**
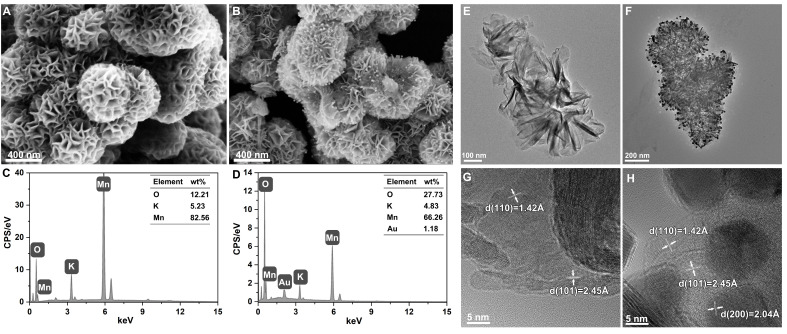
Characterization of MnO_2_ nanoflowers and MnO_2_-Au nanoflowers. (**A**,**B**) SEM image of MnO_2_ nanoflowers (**A**) and MnO_2_-Au nanoflowers (**B**); (**C**,**D**) EDS spectra of MnO_2_ nanoflowers (**C**) and MnO_2_-Au nanoflowers (**D**); (**E**,**F**) TEM images of MnO_2_ nanoflowers (**E**) and MnO_2_-Au nanoflowers (**F**); and (**G**,**H**) HRTEM images of MnO_2_ nanoflowers (**G**) and MnO_2_-Au nanoflowers (**H**).

SEM observations and EDS analyses were employed to investigate the morphology and elemental contents of the prepared MnO_2_ and MnO_2_-Au nanoflowers. As shown in Figure 1A,B, both exhibit a flower-like morphology and similar particle dimensions, with an average size of approximately 580 nm for MnO_2_ and 540 nm for MnO_2_-Au. In addition, it was evident that Au NPs were deposited on the surface of nanoflowers in MnO_2_-Au, with Au NPs showing a monodisperse spherical morphology with a diameter of approximately 22.81 nm (Figure S1) and uniformly dispersed on MnO_2_. In addition, the zeta potentials of MnO_2_ and MnO_2_-Au were −21.53 mV and −21.83 mV (Figure S2), respectively. This result indicated that the zeta potentials of the two nanoflowers were similar, suggesting that the introduction of Au nanoparticles will not affect the stability of the nanoflower. Furthermore, the representative EDS spectrum shown in Figure 1C,D confirmed the presence of K and Mn in both nanoflowers. The presence of elemental Au was detected, as expected in the MnO_2_-Au group. Next, TEM and high-resolution TEM (HRTEM) images were recorded to observe the microstructures of MnO_2_ and MnO_2_-Au nanoflowers. Both exhibited a typical nanosheet stacked structure (Figure 1E,F), which is in good agreement with previous reports [22,26], and the nanosheet stacking structure is assumed to be responsible for forming the flower-like structure [27]. In addition, Au particles could be uniformly distributed and decorated on the nanoflower’s surface (Figure 1F). The HRTEM images, as shown in Figure 1G,H, showed the lattice spacing of MnO_2_ (1.42 Å and 2.45 Å), which are considered the interplanar distances (110) and (101) of δ-MnO_2_, respectively [28]. Moreover, the (200) interplanar distance of Au with a spacing of 2.04 Å was observed in MnO_2_-Au (Figure 1H). These results indicate that two types of MnO_2_ and MnO_2_-Au stacked nanoflowers were obtained.

### 3.2. Evaluation of the Catalase-Mimicking Activity

**Figure 2:**
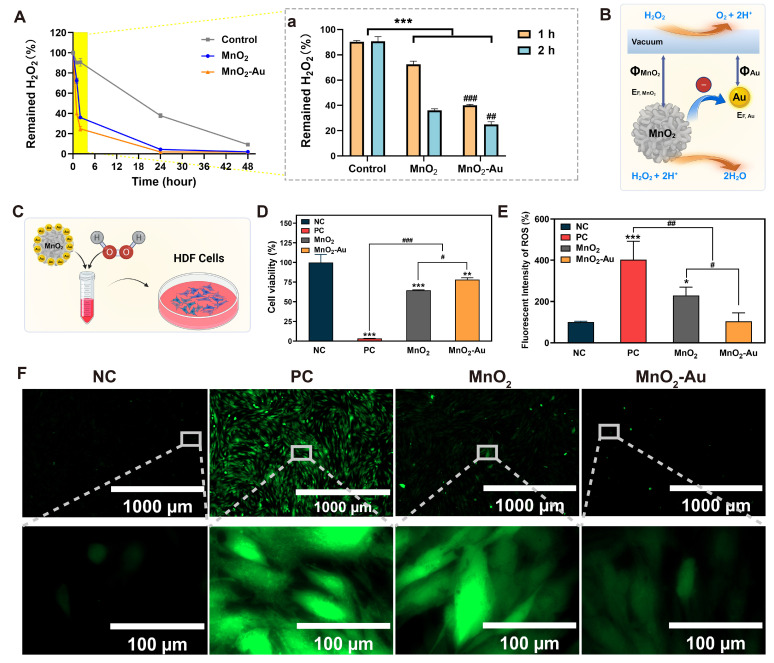
Hydrogen peroxide cleavage and cellular ROS scavenging properties of MnO_2_ and MnO_2_-Au nanoflowers. (**A**) Time-dotted line plot of remaining H_2_O_2_ (%) within 48 h (n = 3), (**a**) The zoom-in of the remaining H_2_O_2_ (%) at 1 and 2 h in (**A**). N = 3, *** *p* < 0.001 indicate significant differences with control; ^##^ *p* < 0.01 and ^###^ *p*< 0.001 indicate significant differences between MnO_2_ and MnO_2_-Au. (**B**) Schematic diagram of MnO_2_-Au nanoflower catalyzed cleavage of H_2_O_2_ (EF: Fermi energy, Φ: work function, as the difference between vacuum energy levels and Fermi energy levels). (**C**) Schematic diagram of the in vitro simulated medium (SM) preparation for cellular assays. (**D**) HDF cell viability treated with the different groups at 24 h. (**E**) Mean fluorescence intensity of oxidative stress in HDF cells after the SM treatment for 30 min. N = 3, * *p* < 0.05, ** *p* < 0.01, and *** *p* < 0.001 indicate significant differences with the NC group; # *p* < 0.05, ## *p* < 0.01 and ### *p* < 0.001 indicate significant differences among PC, MnO_2_, and MnO_2_-Au groups. (**F**) Representative images of fluorescent images for oxidative stress in HDF cells after 30 min of SM treatment.

After the preparation of MnO_2_ and MnO_2_-Au nanoflowers, we focused on the scavenging ability of the two particles for ROS. As hydrogen peroxide is a product of the superoxide anion radical (·O_2_^−^) and is an active ROS [29], the H_2_O_2_ elimination properties of both particles within 48 h were evaluated. As shown in Figure 2A, a significant reduction in H_2_O_2_ was induced in both the MnO_2_ and MnO_2_-Au groups. The concentration of H_2_O_2_ significantly decreased during the initial 2 h after treatment with both particles compared with the control (Figure 2A(a)). Thus, MnO_2_-Au nanoflowers significantly promoted H_2_O_2_ elimination, especially in the initial 2 h of the reaction, with hydrogen peroxide residual ratios of 40.03 ± 0.60% after 1 h and 24.84 ± 1.85% after 2 h. This involved more efficient catalytic cleavage than that of MnO_2_ with rates of 72.53 ± 2.02% (1 h) and 36.05 ± 1.05% (2 h). In contrast, the residual H_2_O_2_ in the control group, which was not treated with any nanoenzymes, was consistently 90.68% within 2 h. Even up to 24 and 48 h, H_2_O_2_ decomposed due to instability in the control group, but the remaining amount was still significantly higher than those of the MnO_2_ and MnO_2_-Au groups. These data confirm that both Mn-based nanoflowers were effective in nanoenzymatic catalyzed cleavage of H_2_O_2_, with MnO_2_-Au nanoflowers superior to MnO_2_ nanoflowers. The more efficient decomposition of hydrogen peroxide by MnO_2_-Au nanoflowers than by MnO_2_ nanoflowers may be because (Figure 2B) MnO_2_ can obtain free electrons to migrate to Au NPs due to the difference in the Fermi levels of the two materials after the combination of MnO_2_ nanoflowers and Au NPs. Moreover, in the process of hydrogen peroxide decomposition, MnO_2_ provides only an oxygen vacuum to play a role, whereas Au can also provide electrons [26]. This increases the carrier lifetime compared with MnO_2_ alone, prevents the compounding of electrons and holes in MnO_2_, and enhances the catalytic ability. The construction of nanocatalytic enzyme composite platforms has been considered using three-dimensional folded nanostructures of MnO_2_ as high-specific surface area platforms for the integration of Au NPs with enhanced electron transfer activity [30,31]. Zhang et al. reported that endogenous hydrogen peroxide scavenging can be effectively achieved using MnO_2_ nanosheet hybrid hydrogels as wound therapy for multidrug-resistant skin infections [21]. In this study, we developed a MnO_2_-based noble metal catalyst with high efficiency and superior stability. Owing to the different Fermi levels, the introduction of Au NPs involves the abundant oxygen vacancy of MnO_2_, enhancing the catalytic performance of MnO_2_-Au nanoflowers. Meanwhile, the electron regulation between MnO_2_ and Au improved the lifetime and long-term stability. Notably, MnO_2_ decorated with Au NPs exhibited high-efficiency endogenous hydrogen peroxide scavenging and effective cellular-protective ability under oxidative stress.

### 3.3. In Vitro ROS Scavenging Activity of MnO_2_ Nanoflowers

Encouraged by the results of the H_2_O_2_-scavenging assay, we performed an in vitro cell catalase-mimicking study of MnO_2_ and MnO_2_-Au nanoflowers. Under diabetic conditions, HDFs help regulate inflammation and cell proliferation by secreting a series of signaling molecules [32]. Because HDFs play a key role in skin regeneration and wound healing, HDF cells were chosen to construct an in vitro catalytic model under the SM model of hydrogen peroxide pre-treated with nanoflowers. The aim was to explore the protective potential of MnO_2_ and MnO_2_-Au on cells under an oxidative stress environment (Figure 2C). As shown in Figure 2D, compared with the NC group, the cell viability in the PC group was only 3.34%, while the cell viability in the MnO_2_ group and MnO_2_-Au group recovered to 64.59% and 78.06%, respectively. Notably, in the MnO_2_-Au group, pre-treatment with H_2_O_2_ for only 1 h ensured cell viability greater than 75%, providing important support for reducing cell damage induced by ROS. However, it is undeniable that the two nanoflowers did not completely restore cell viability to the level of the untreated control group, which may be due to the residual H_2_O_2_. It is also consistent with the results of the catalase-mimicking activity of nanoflowers (Figure 2A(a)). To further investigate the protective effects of nanoflowers against cellular oxidative stress, we evaluated the in vitro antioxidant properties of MnO_2_ and MnO_2_-Au nanoflowers by employing a ROS-sensitive dichlorofluorescent probe to detect intracellular ROS changes [33]. As shown in Figure 2E, the PC group led to a ~4-fold increase in the level of ROS compared to the NC group, while the MnO_2_-Au group had no significant difference in the level of ROS compared to the NC group (Figure 2E), suggesting that MnO_2_-Au effectively exerts the function of nanoenzymes to protect HDFs from oxidative stress damage.

In addition, ROS fluorescence results confirmed that the HDF cells showed a large area of green fluorescence in the PC group, suggesting that the cells were under severe oxidative stress. In contrast, the fluorescence intensities of the two groups treated with nanoflowers were significantly weakened, with almost no ROS expression observed in the MnO_2_-Au group (Figure 2F). All these results indicated that MnO_2_-Au nanoflowers could efficiently scavenge ROS. The prominently lower fluorescence intensity in the presence of MnO_2_-Au than in the presence of the MnO_2_ group indicates an elevated ROS catalytic capacity due to the effective electron modulation of the Au NPs [34,35].

### 3.4. HDF Cell Migration and Collagen I Expression Assay

**Figure 3:**
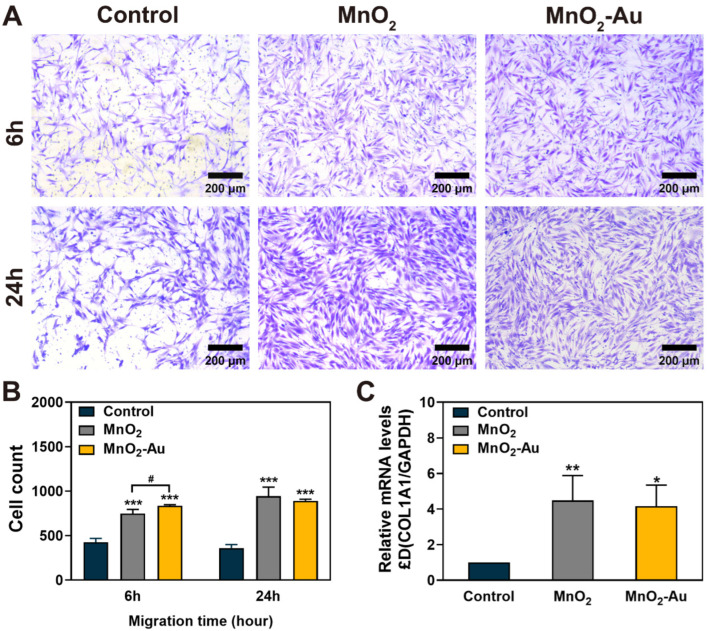
Cell migration and *COL1A1* expression of HDFs cultured with MnO_2_ and MnO_2_-Au nanoflowers. (**A**,**B**) Cell migration of HDFs at 6 h and 24 h, and (**C**) *COL1A1* expression levels in HDFs at 24 h; n = 3, * *p* < 0.05, ** *p* < 0.01, *** *p* < 0.001 indicate significant differences with control; # *p* < 0.05 indicates significant differences between MnO_2_ and MnO_2_-Au.

Collective cell migration is critical in skin regeneration and wound healing and is directly related to subsequent ECM remodeling, collagen regeneration, and wound contraction [36]. Evaluating the properties of pro-cell migration under oxidative stress is essential to determine the potential of nanoflowers for wound repair. A transwell migration system was applied to assess the effect of nanoflowers on HDF cell migration on a three-dimensional scale. As shown in Figure 3A, a higher proportion of HDF cells migrated at 6 and 24 h in the MnO_2_ and MnO_2_-Au treatment groups than in the control group. Quantitative analysis (Figure 3B) showed that HDF cell migration was inhibited under H_2_O_2_ treatment. In contrast, MnO_2_ and MnO_2_-Au treatment effectively increased the number of migrating HDF cells, suggesting that alleviating the ROS burden is necessary and critical to initiating subsequent tissue repair. It should be noted that since the HDF cell migration experimental system (including the volume of medium, the setup of the upper and lower chambers, and the cell seeding density) differs from that of the cell viability assay, which resulted in a more significant amount of cell migration than the cell viability results. In general, our results confirmed that MnO_2_-Au nanoflowers can significantly enhance the migration of HDF cells to the lower chamber under the effective cleavage of H_2_O_2_. In addition, the results of collagen I expression on HDF cells (Figure 3C) showed that both nanoflowers could effectively enhance the expression of *COL1A1* in damaged HDF cells compared to the H_2_O_2_-treated group. It is well-known that enhancing the ability of HDFs to secrete COL-I is fundamental to repairing damaged dermal tissues [37].

### 3.5. Characterization of MnO_2_ and MnO_2_-Au Nanoflower Composite Hydrogels

**Figure 4:**
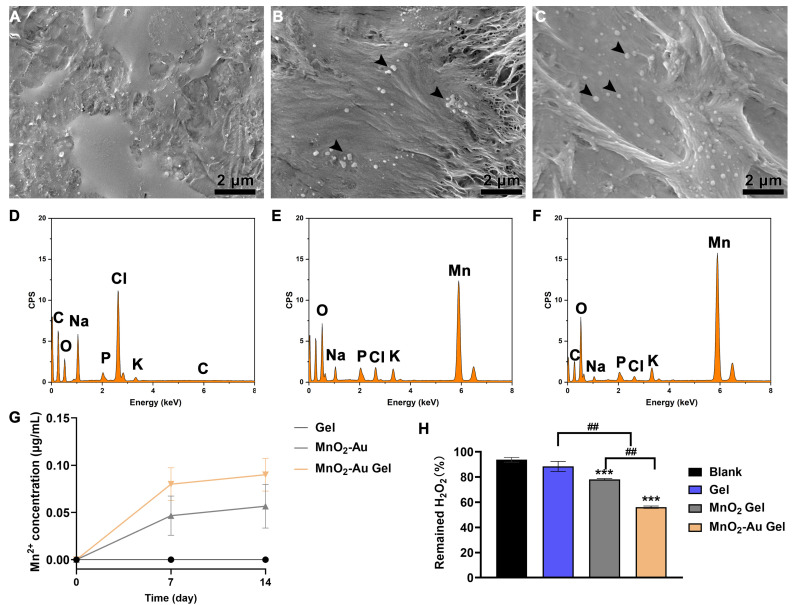
Characterization and properties of MnO_2_ and MnO_2_-Au nanoflowers composite hydrogels. (**A**–**C**) SEM images of gel (**A**), MnO_2_ gel (**B**), and MnO_2_-Au gel (**C**), the black arrow points to the nanoflowers. (**D**) EDS profiles of the gel, (**E**,**F**) EDS profiles of particles in MnO_2_ gel (**E**) and MnO_2_-Au gel (**F**). (**G**) The concentration of Mn ions released in gel at 7 and 14 days. (**H**) Hydrogen peroxide cleavage after the treatment for 2 h. N = 3, *** *p* < 0.001 indicate significant differences with blank; ^##^ *p* < 0.01 indicates significant differences among gel, MnO_2_ gel, and MnO_2_-Au gel.

To guarantee the nanoflowers can operate on a wound surface, we selected the hydrogel to composite the two nanoflowers, and images of the constructed composite hydrogels are shown in Figure S3. Furthermore, SEM images and EDS patterns (Figure 4A–F, Table S1) showed that the nanoflowers were uniformly distributed on the surface of the hydrogel. The ICP-MS results (Figure 4G) showed that elemental Mn could be detected in the hydrogel immersed in PBS, which suggests that the nanoflowers can be released from the hydrogel with time. A further hydrogen peroxide decomposition test revealed that the gel alone could not effectively cleave H_2_O_2_. Moreover, the composite nanoflower hydrogels could effectively act as nanoenzymes to catalyze H_2_O_2_ cleavage (Figure 4H). In addition, no significant cytotoxicity was observed in any of the three hydrogel groups compared with the blank group (Figure S4). The above results provided a foundation for subsequent in vivo experiments.

### 3.6. In Vivo Wound Regeneration

**Figure 5:**
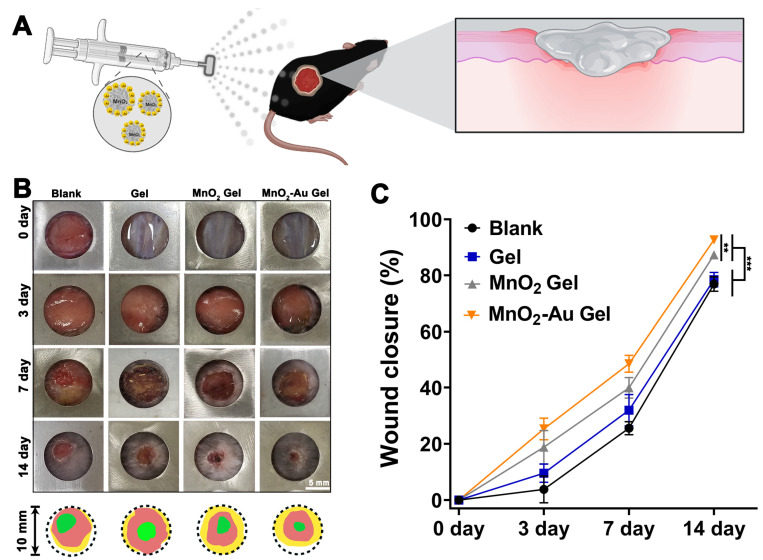
Genal observation of MnO_2_ and MnO_2_-Au composite hydrogels promoting wound healing in diabetic mice. (**A**) Schematic diagram of the skin defect model. (**B**) Images of wounds at days 0, 3, 7, and 14 and wound overlap maps, scale bar: 5 mm. (**C**) Wound closure (%) within 14 days. N = 4, ** *p* < 0.01, *** *p* < 0.001 between the different treatment at day 14.

To further evaluate the in vivo healing activity in diabetic wounds, a diabetic mouse model with full-thickness injuries was employed (Figure 5A). Wound healing was recorded for each group at predetermined time points. As shown in Figure 5B,C, all groups showed varying degrees of wound healing on the 14th postoperative day, with the MnO_2_-Au gel group showing the most efficient wound closure. On day 14, the wounds in the blank and gel groups were not yet completely crusted, which may be related to the high oxidative stress from diabetes that impedes the regeneration process [38].

**Figure 6:**
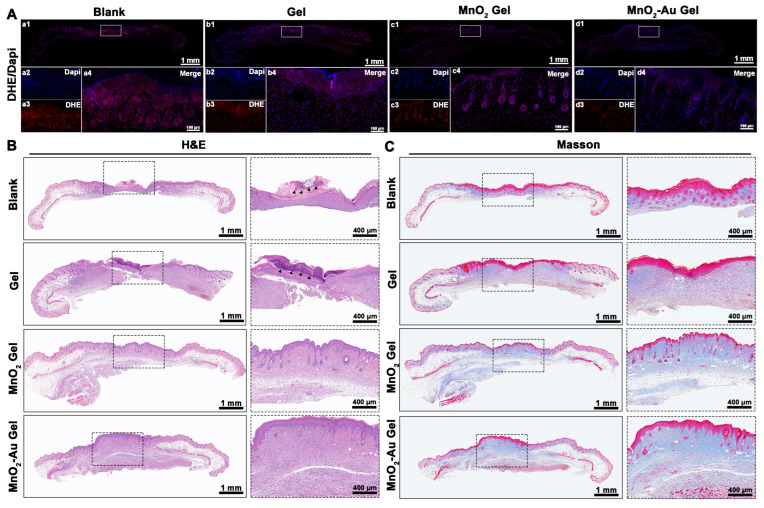
DHE and histological observation of MnO_2_ and MnO_2_-Au composite hydrogels promoting wound healing in diabetic mice. (**A**) DHE fluorescence staining of wound tissue on day 14, (**a2**–**a4**, **b2**–**b4**, **c2**–**c4**, **d2**–**d4**) is the 40× magnification image corresponding to the white box in (**a1**, **b1**, **c1**, **d1**). (**B**) H&E and (**C**) Masson staining for each group on day 14, the dashed box presented at 10× magnification. The arrow points to the area of inflammatory infiltration.

To evaluate ROS levels within the tissues, we stained skin tissue sections using DHE and 4′,6-diamidino-2-phenylindole [4,39]. Figure 6A shows that the intensity of red fluorescence representing ROS levels in skin tissues in the blank group was significantly higher than that in the other three groups. The intensities of ROS expression in tissues of the MnO_2_ gel and MnO_2_-Au gel groups remained low, especially in the MnO_2_-Au gel group, in which almost no evident reactive expression was observed. This suggested that MnO_2_-Au effectively reversed the local high reactive oxygen species microenvironment. This also agrees with our in vitro results (Figure 2).

The detailed characteristics of diabetic wound healing under MnO_2_ and MnO_2_-Au nanoflower composite gel treatments were assessed using H&E and Masson’s staining (Figure 6B,C). Scattered inflammatory cell infiltration tissue necrosis resulting from crusting was observed in the epidermal layer in the blank and gel groups using H&E staining. In contrast, inflammatory cells had minimal infiltration and an intact epidermal layer in the MnO_2_ gel and MnO_2_-Au gel groups. The reduced level of inflammation may be attributed to relieving oxidative stress by MnO_2_ and MnO_2_-Au nanoflowers, which accelerated the transition of the wound from the inflammatory to the proliferative phase [40]. In addition, the MnO_2_-Au group showed superior healing to the other groups (Figure 6B). Furthermore, Masson’s staining could assess the newly deposited collagen and keratin, where collagen was stained blue, and keratin was stained red. As shown in Figure 6C, a striped keratin-positive layer was found in all groups. The collagen deposition was more abundant, extensive, and well-organized under MnO_2_-Au nanoflower treatment (Figure 6C). In addition, by analyzing the percentage of the collagen deposition area, the results showed that the MnO2-Au gel groups were able to enhance the collagen content compared to the other three groups (Figure S5), indicating that MnO2-Au effectively promotes high-quality healing of skin tissues. It is known that the wound-healing process can be divided into four overlapping stages: hemostasis, inflammation, hyperplasia, and remodeling [41,42]. One of the most critical stages for wound healing in diabetes is managing the inflammatory phase. Usually, the inflammatory phase lasts about 3~7 days and is accompanied by an inflammatory response that causes vasodilation and increased capillary permeability. However, diabetic wounds are difficult to heal in a well-organized and timely manner as normal wounds, mainly due to an imbalance in pro-inflammatory factor regulation, increased ROS, and excessive protease production [43]. Excessive ROS can disrupt the redox balance of cells, damaging the ECM and cell membranes, thereby hindering wound healing. In our study, MnO2-Au nanoflowers have shown excellent peroxide scavenging activity, which may scavenge excessive ROS under the high oxidative stress microenvironment, thereby exhibiting significant advantages in promoting high-quality healing of skin tissues in diabetes.

## 4. Conclusions

In summary, we have successfully synthesized MnO_2_ nanoflowers decorated with Au NPs using a hydrothermal reaction. We systematically investigated the catalytic ability of the MnO_2_-Au nanoflowers and their potential for treating chronic wounds. It was observed that MnO_2_-Au nanoflowers were able to cleave hydrogen peroxide, demonstrating rapid and high catalytic efficiency. MnO_2_-Au nanoflowers reduced the intracellular oxidative stress response of HDF cells via the nanoenzymatic cleavage of hydrogen peroxide, showing its superior antioxidant protective ability to enhance migration and Col-I expression of the oxidative stress state of HDF cells. In the in vivo diabetic wound experiments, MnO_2_-Au nanoflowers composite hydrogels effectively enhanced the wound closure rate and exhibited extensive collagen deposition. Our findings highlight that MnO_2_-Au nanoflowers exhibit potential application as nanoenzyme delivery systems for diabetic wound healing, achieving a promising strategy featuring biosafety, excellent stability, and high efficiency in antioxidant-associated treatment.

## Figures and Tables

**Table 1:** The sequences for HDFs PCR primers.

Gene		Primer Sequence
*COL1A1*	Forward	5′-GAGGGCCAAGACGAAGACATC-3′
	Reverse	5′-CAGATCACGTCATCGCACAAC-3′
*GAPDH*	Forward	5′-ACAACTTTGGTATCGTGGAAGG-3′
	Reverse	5′-GCCATCACGCCACAGTTTC-3′

## Data Availability

The original contributions presented in this study are included in the article; further inquiries can be directed to the corresponding author.

## Supplementary Materials

The following supporting information can be downloaded at: https://www.mdpi.com/article/10.3390/pharmaceutics16101244/s1. Figure S1: Size distribution of Au NPs on the MnO_2_-Au nanoflowers. Figure S2: Zeta-potential of MnO_2_ and MnO_2_-Au nanoflowers. Figure S3: Photographic images of MnO_2_ and MnO_2_-Au nanoflowers composite gel. (A) Gel, (B) MnO_2_ Gel, (C) MnO_2_-Au Gel. Figure S4: Cell viability on exposure to MnO_2_ and MnO_2_-Au composite gel after 24 h exposure. Figure S5: Statistical analysis of the relative area covered by collagen in the regenerated tissue calculated on the basis of Masson trichrome staining. n = 4. ** *p* < 0.01, and *** p < 0.001 indicate significant differences with Blank; # *p* < 0.05 indicates significant differences among Gel, MnO_2_ Gel, and MnO_2_-Au Gel. Table S1. Chemical compositions of Gel, MnO_2_ Gel, and MnO_2_-Au Gel correspond to EDS spectra.
