# Supplementary material for: Using Hybrid MnO2-Au Nanoflowers to Accelerate ROS Scavenging and Wound Healing in Diabetes
Source: Pharmaceutics. 2024 Sep 25;16(10):1244. doi: 10.3390/pharmaceutics16101244 (PMC11509962; doi:10.3390/pharmaceutics16101244)
Supplement: Supplementary file 1 [file pharmaceutics-16-01244-s001.zip › pharmaceutics-3184306-supplementary.pdf]

Supplementary Material

Using Hybrid MnO_2-Au Nanoflowers to Accelerate ROS Scavenging and Wound Healing in Diabetes

Ning Jiang [1,†], Xinwei Liu [2,†], Baiyan Sui [2], Jiale Wang [3,4], Xin Liu [2,*] and Zun Zhang [5,*]

[1] Department of Oral and Craniomaxillofacial Science, Shanghai Key Laboratory of Stomatology, Shanghai Ninth People's Hospital, Shanghai Jiao Tong University School of Medicine; College of Stomatology, Shanghai Jiao Tong University, Shanghai 200011, China

[2] Department of Dental Materials, Shanghai Biomaterials Research & Testing Center, Shanghai Ninth People's Hospital, Shanghai Jiao Tong University School of Medicine; College of Stomatology, Shanghai Jiao Tong University; National Center for Stomatology; National Clinical Research Center for Oral Diseases; Shanghai Key Laboratory of Stomatology, Shanghai 200011, China

[3] College of Science, Donghua University, Shanghai 201620, China

[4] Shanghai Institute of Intelligent Electronics and Systems, Donghua University, Shanghai 201620, China

[5] Department of Stomatology, Shanghai East Hospital, Tongji University, Shanghai 200120, China

† The authors contributed equally to this work.

* Correspondence: zzhang5609@163.com (Z. Zhang), liuxin0556@163.com (X. Liu)

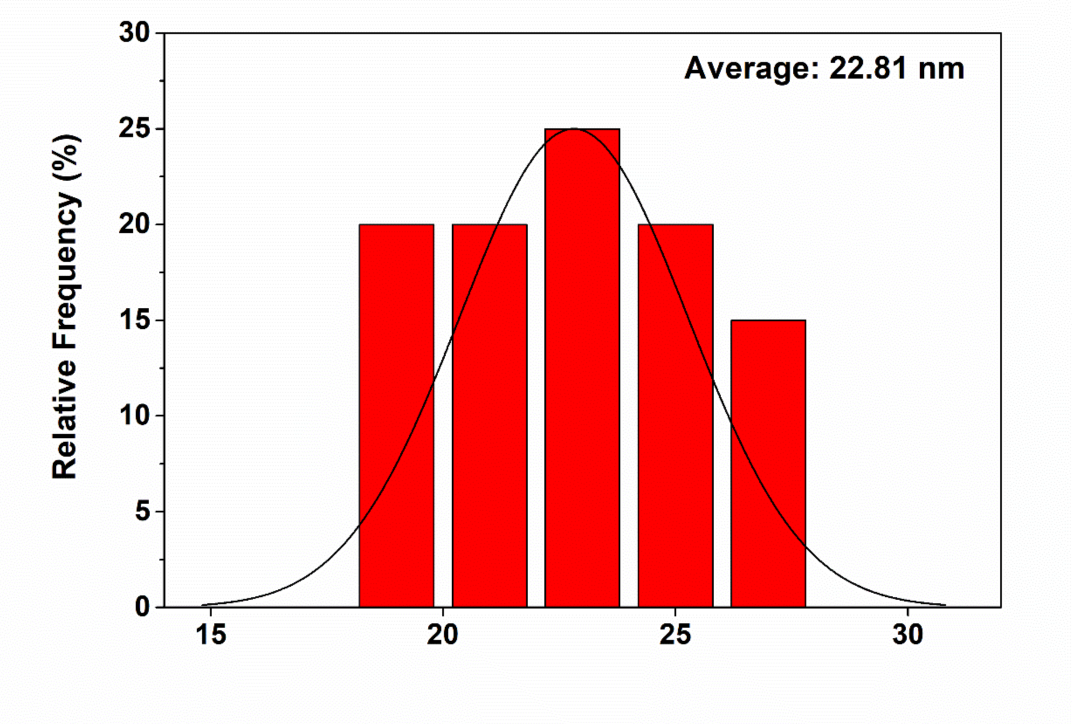

Figure S1. Size distribution of Au NPs on the MnO_2-Au nanoflowers.

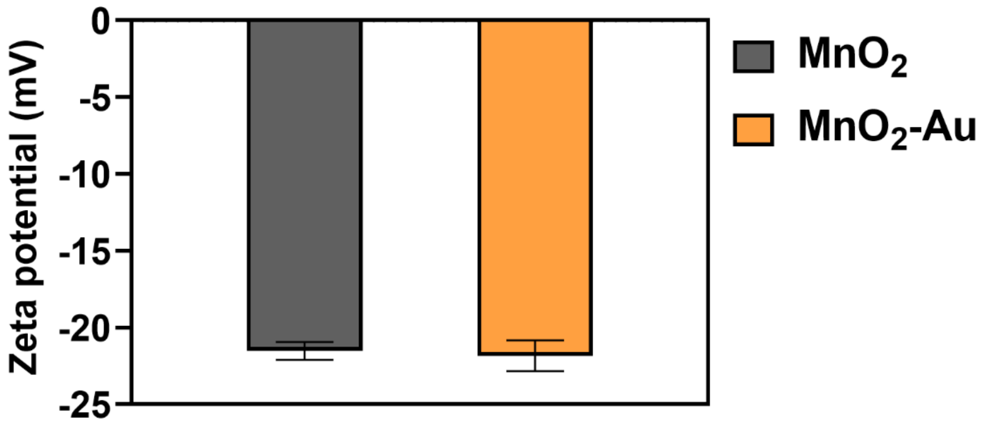

Figure S2. Zeta-potential of MnO2 and MnO2-Au nanoflowers.

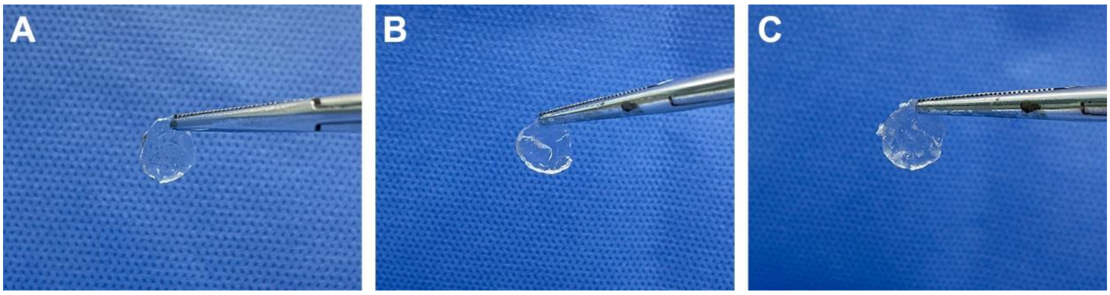

Figure S3. Photographic images of MnO_2 and MnO_2-Au nanoflowers composite gel. (**A**) Gel, (**B**) MnO_2 Gel, (**C**) MnO_2-Au Gel.

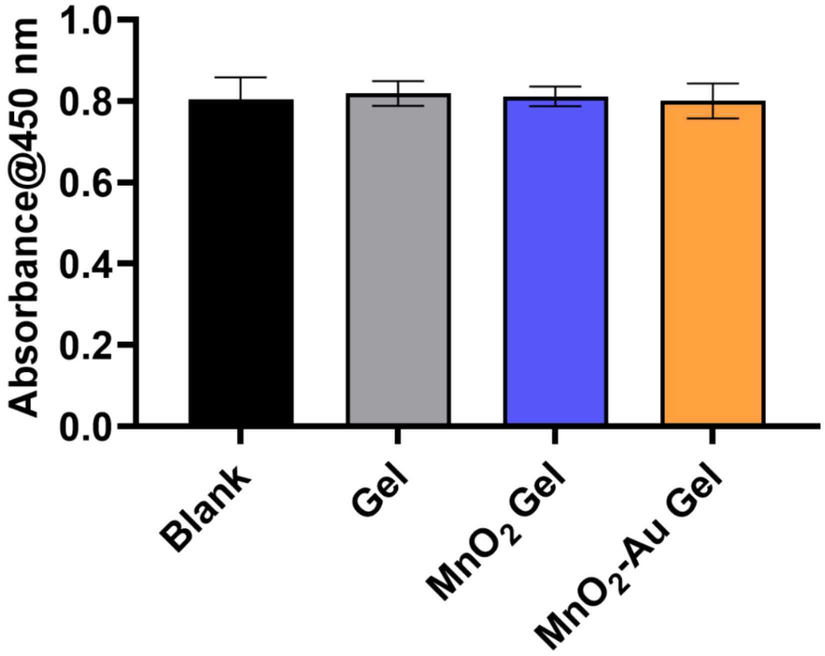

Figure S4. Cell viability on exposure to MnO_2 and MnO_2-Au composite gel after 24 h exposure.

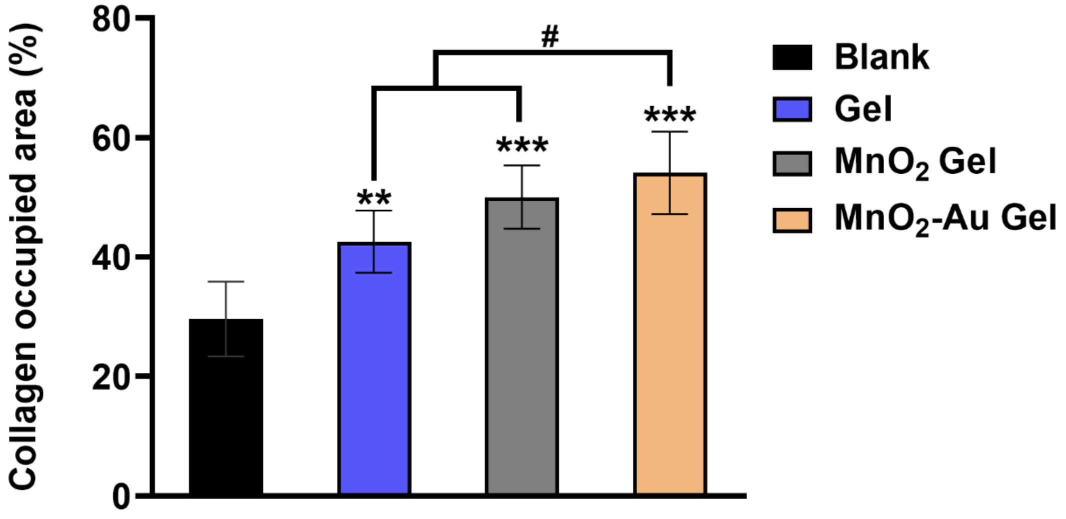

Figure S5. Statistical analysis of the relative area covered by collagen in the regenerated tissue calculated on the basis of Masson trichrome staining. n=4. ** P < 0.01, and *** P < 0.001 indicate significant differences with Blank; #P< 0.05 indicates significant differences among Gel, MnO2 Gel, and MnO2-Au Gel.

Table S1. Chemical compositions of Gel, MnO_2 Gel, and MnO_2-Au Gel correspond to EDS spectra.

Element / Group	Weight %						
	**Mn**	**O**	**C**	**K**	**Na**	**Cl**	**P**
**Gel**	**0.0**	**18.2**	**59.1**	**0.7**	**7.8**	**13.4**	**0.7**
**MnO_2 Gel**	**46.3**	**27.4**	**18.0**	**3.7**	**1.9**	**1.8**	**0.9**
**MnO_2-Au Gel**	**63.7**	**16.8**	**14.4**	**2.4**	**1.5**	**0.6**	**0.5**
